# Ciprofloxacin-Induced Hepatocellular Injury in an Elderly Patient With Polypharmacy and Multiple Comorbidities

**DOI:** 10.7759/cureus.104858

**Published:** 2026-03-08

**Authors:** Ritika Chamlagai, Aysha Tahsin, Dania Halperin, Richard Weinberger

**Affiliations:** 1 Internal Medicine, Philadelphia College of Osteopathic Medicine, Philadelphia, USA; 2 Physical Medicine and Rehabilitation, The Wright Center for Graduate Medical Education, Scranton, USA; 3 Internal Medicine, The Wright Center for Graduate Medical Education, Scranton, USA

**Keywords:** acute kidney injury, acute liver injury, antibiotic adverse effects, ciprofloxacin, drug-induced liver injury, fluoroquinolone hepatotoxicity, inpatient rehabilitation, polypharmacy in elderly, rhabdomyolysis-associated transaminitis

## Abstract

Ciprofloxacin is a widely prescribed fluoroquinolone antibiotic that may cause transient aminotransferase elevations, although clinically significant drug-induced liver injury remains rare. We present a 72-year-old woman with multiple comorbidities who developed worsening transaminitis shortly after ciprofloxacin initiation for a urinary tract infection. The patient was recently hospitalized for rhabdomyolysis-associated acute kidney injury with improving liver enzymes prior to antibiotic exposure. After four doses of ciprofloxacin, alanine aminotransferase and aspartate aminotransferase increased, while bilirubin remained normal. Given the temporal association and absence of alternative hepatic pathology, ciprofloxacin-induced hepatocellular injury was suspected. Discontinuation of the medication resulted in prompt improvement in liver enzymes. This case highlights the importance of recognizing ciprofloxacin-associated hepatotoxicity, particularly in medically complex patients, and emphasizes early discontinuation to prevent progression of liver injury.

## Introduction

Ciprofloxacin is a widely prescribed fluoroquinolone antibiotic used in the treatment of common bacterial infections, including urinary tract infections and pneumonia [1]. Although it is generally well tolerated, commonly reported adverse effects include nausea, vomiting, and diarrhea, while more serious complications such as tendinitis, peripheral neuropathy, and central nervous system toxicity have also been documented [2]. Hepatotoxicity associated with ciprofloxacin is considered uncommon, with transient serum aspartate aminotransferase (AST) elevations occurring in approximately 1-3% of patients during therapy [1]. Nevertheless, clinically significant drug-induced liver injury (DILI) has been described in multiple case reports demonstrating variable biochemical and clinical presentations. For example, an 84-year-old patient treated with ciprofloxacin for a urinary tract infection developed a rash followed by delayed jaundice and marked elevations in AST, alkaline phosphatase (ALP), and bilirubin (total bilirubin 8.2 mg/dL) after discontinuation of therapy, with resolution over six weeks [1]. In another case, a 37-year-old woman treated for uncomplicated pyelonephritis similarly developed elevations in AST, ALT, and ALP that returned to baseline after switching to ceftriaxone [3]. Additional reports include a 74-year-old woman who presented with weakness, jaundice, and elevated AST, ALT, ALP, and bilirubin after high-dose ciprofloxacin therapy for a urinary tract infection [4]. Lastly, a 32-year-old woman developed acute cholestatic liver injury after only three days of ciprofloxacin exposure [5]. Collectively, these cases highlight the unpredictable onset, diverse biochemical patterns, and generally reversible course of ciprofloxacin-induced liver injury reported in the past. Here, we describe another case of acute DILI due to ciprofloxacin administration. 

## Case presentation

A 72-year-old woman with a history of hypertension, hyperlipidemia, type 2 diabetes mellitus, hypothyroidism, and obstructive sleep apnea presented with several days of nausea, vomiting, decreased urine output, and progressive generalized weakness. Family members also reported worsening cognitive decline over the preceding three months. Her home medications included meloxicam and losartan, and donepezil had been initiated 10 days prior for dementia.

Initial laboratory evaluation demonstrated acute kidney injury and transaminitis, with markedly elevated creatine kinase and myoglobin levels consistent with rhabdomyolysis (Table 1). Urinalysis showed positive blood with minimal red blood cells, mild proteinuria, and trace leukocyte esterase. Nephrology suspected prerenal azotemia versus ischemic acute tubular necrosis in the setting of volume depletion and impaired renal autoregulation related to concurrent angiotensin receptor blocker and NSAID use. Rheumatologic evaluation, including inflammatory markers and muscle biopsy, was unremarkable, and the liver enzyme elevation was attributed to muscle injury. The patient was treated with intravenous fluids, resulting in improvement in renal function and downtrending creatine kinase levels. She was subsequently transferred to our inpatient rehabilitation facility for continued recovery.

**Table 1:** Laboratory values during hospitalization and after ciprofloxacin exposure

Laboratory Test	Reference Range	Initial Admission	Pre-ciprofloxacin (Rehab Admission)	After Four Doses of Ciprofloxacin	After Discontinuation of Ciprofloxacin
Blood Urea Nitrogen (mg/dL)	7–20	68	31–28	35	35
Creatinine (mg/dL)	0.6–1.3	4.9	2.3	2.5	2.0
AST (U/L)	10–40	270	46	193	64
ALT (U/L)	7–56	252	163	274	189
Creatine Kinase (U/L)	30–170	>17,000 (peak)	1,289 (at discharge)	—	—
Myoglobin (ng/mL)	<85	>4,000	—	—	—
Total Bilirubin (mg/dL)	0.2–1.2	Normal	Normal	Normal	Normal

During rehabilitation, the patient developed new-onset, non-radiating, crampy left-sided abdominal and flank pain associated with fever and chills. Urinalysis and urine culture were consistent with a urinary tract infection due to pan-sensitive Escherichia coli, and she was started on oral ciprofloxacin 500 mg daily. After four doses of ciprofloxacin, repeat laboratory testing demonstrated worsening transaminitis compared to pre-treatment values, while total bilirubin remained within normal limits (Table 1). Renal function also showed mild worsening during this period.

Due to concern for DILI and possible renal function deterioration, ciprofloxacin was discontinued. Subsequent laboratory evaluation demonstrated improvement in liver enzymes, while renal function remained stable to slightly improved (Table 1).

While additional details such as baseline renal function, renal imaging studies, and the exact etiology of rhabdomyolysis were limited in the available documentation, these factors did not change the clear temporal relationship between ciprofloxacin initiation and worsening liver enzyme elevation.

## Discussion

DILI is one of the leading causes of acute hepatic failure in Western countries and poses a significant diagnostic challenge, particularly in elderly patients with polypharmacy and multiple comorbidities [4]. Fluoroquinolones are widely prescribed antimicrobial agents and are generally well tolerated. However, they have increasingly been recognized as rare causes of hepatotoxicity. Ciprofloxacin, one of the most commonly prescribed fluoroquinolones for urinary tract infections, has been associated with clinically significant but uncommon liver injury [6]. This case highlights the diagnostic complexity of ciprofloxacin-associated hepatotoxicity in a medically complex patient with concurrent renal injury due to rhabdomyolysis and recent exposure to multiple potentially hepatotoxic medications.

Fluoroquinolone exposure has been associated with increased risk of liver injury. A Veterans Affairs case-control study demonstrated increased hepatotoxicity risk with ciprofloxacin use, and a nationwide Swedish cohort study reported a twofold increased risk of acute liver injury, although the absolute risk remained low [7,8]. Despite its rarity, ciprofloxacin-associated hepatotoxicity remains clinically relevant due to widespread utilization.

The mechanism underlying ciprofloxacin-associated hepatotoxicity remains incompletely understood. Current evidence suggests that fluoroquinolone-induced liver injury is primarily idiosyncratic and may involve mitochondrial dysfunction, oxidative stress, and immune-mediated hepatocellular injury [9]. Experimental studies have demonstrated that fluoroquinolones can impair mitochondrial DNA replication and increase reactive oxygen species, leading to hepatocyte injury and apoptosis in susceptible individuals [9].

The diagnosis of ciprofloxacin-induced liver injury remains challenging due to the absence of specific diagnostic biomarkers and the need to exclude alternative etiologies. Published reports frequently utilize causality assessment tools such as the Roussel Uclaf Causality Assessment Method (RUCAM), WHO-Uppsala Monitoring Centre (WHO-UMC) causality assessment, and Naranjo scale to support drug attribution [4]. These assessments rely on factors such as temporal relationships, improvement following drug discontinuation, and exclusion of other causes of liver injury. In previously reported cases, patients demonstrated improvement in liver enzyme abnormalities following cessation of ciprofloxacin and exclusion of competing etiologies including viral hepatitis, autoimmune disease, and obstructive hepatobiliary disease [4,6]. Similarly, in this case, liver enzyme levels worsened following ciprofloxacin initiation and improved upon discontinuation, supporting ciprofloxacin as the possible offending agent. The Naranjo Score for this case was +4, which suggests that the reaction is possibly due to ciprofloxacin administration [1]. 

This case was further complicated by the patient’s recent hospitalization for rhabdomyolysis, which is known to cause elevations in aminotransferases due to muscle injury. Distinguishing hepatic from muscular causes of transaminitis represents a recognized diagnostic challenge. In this patient, improvement in creatine kinase levels and liver enzyme abnormalities prior to ciprofloxacin exposure, followed by recurrent transaminitis after antibiotic initiation, supports a secondary hepatic insult rather than persistent muscle injury.

Although the absolute risk of ciprofloxacin-induced hepatotoxicity is low, the widespread use of fluoroquinolones magnifies the clinical impact of this adverse effect. Previous literature emphasizes that early recognition and prompt discontinuation of the offending agent are critical for improving patient outcomes. Delayed recognition or inadvertent re-exposure has been associated with worsening liver injury and fatal hepatic failure [4]. This case reinforces the importance of maintaining clinical suspicion for antibiotic-associated hepatotoxicity when new liver enzyme abnormalities occur shortly after medication initiation.

This case contributes to the existing literature by demonstrating ciprofloxacin-associated hepatotoxicity in a patient with recent rhabdomyolysis and acute kidney injury, highlighting the difficulty of distinguishing multifactorial causes of transaminitis in medically complex patients. It further underscores the importance of careful medication reconciliation and monitoring of hepatic function in patients receiving fluoroquinolone therapy, particularly those with multiple comorbidities or recent hepatic or muscular injury.

## Conclusions

Ciprofloxacin-induced liver injury is an uncommon but clinically important adverse effect that can occur shortly after initiation of therapy. This case highlights the diagnostic challenges of identifying drug-induced hepatocellular injury in elderly patients with polypharmacy, recent rhabdomyolysis, and concurrent renal dysfunction. The temporal relationship between ciprofloxacin exposure and worsening transaminitis, followed by improvement after drug discontinuation, supports ciprofloxacin as the likely offending agent. Given the widespread use of fluoroquinolones, clinicians should maintain a high index of suspicion for hepatotoxicity when new liver enzyme abnormalities develop during treatment. Early recognition, prompt discontinuation of the suspected medication, and careful monitoring of liver function are essential to prevent progression of liver injury and improve patient outcomes.
